# Nutritional Habits and Recommendations in the COVID-19 Era

**DOI:** 10.3390/nu14030693

**Published:** 2022-02-07

**Authors:** Antonis Zampelas

**Affiliations:** Laboratory of Dietetics and Quality of Life, Department of Food Science and Human Nutrition, School of Food and Nutritional Sciences, Agricultural University of Athens, 11855 Athens, Greece; azampelas@aua.gr

The most recent coronavirus outbreak is that of 2019 SARS-CoV-2, which causes the Coronavirus Disease-2019 (COVID-19). On 30 January 2020, the World Health Organization declared a warning worldwide regarding this new outbreak, which started in Wuhan, a city located in China. On 24 February 2020, WHO acknowledged that the SARS-CoV-2 had the potential to spread worldwide and that subsequently could become a pandemic.

Since then, the academic community has published numerous papers with the primary aim not only to understand how to treat the disease but also to investigate ways to prevent it. It has been suggested that people who are malnourished and have impaired immune system are more likely to be infected and to experience more severe symptoms. Furthermore, lockdowns and prolonged uncertainties have had an impact on nutritional habits.

*Nutrients* recently published a very interesting paper that investigated changes in dietary habits and associated practices, as well as in physical activity, during the COVID-19 pandemic and compared them with the ones before the onset of lockdowns in three European countries: Poland, Austria, and the United Kingdom [1]. The major findings of this cross-sectional study are noteworthy because they indicate changes that could affect nutritional status and health indices, which in turn could undermine the function of the immune system and its ability to act efficiently against external pathogen invasions, including SARS-CoV-2. In particular, results from this study indicated that changes in work and in general working conditions, or even unemployment, could have led to an increase in alcohol consumption. Additionally, physical activity levels were reported to be significantly decreased, which subsequently may have adversely affected body mass. Furthermore, although an increased interest in online grocery shopping was observed, a decrease in shopping frequency was also reported. It was also pointed out that the participants were more often consuming self-prepared meals and ordering readymade meals from restaurants or catering companies. On the other hand, the lockdown decreased restaurant attendance, and the frequency of eating out, in all the three countries studied. Moreover, an increased frequency of the daily consumption of food products such as dairy, grains, fats, vegetables, and sweets was observed, as well as an increase in snacking and in the frequency of purchasing frozen goods and food with long shelf life. Finally, the consumption of 4–5 meals a day, ordering readymade meals at restaurants, and increasing the frequency of sweets, fruits, and alcohol consumption, increased the odds of gaining body mass.

The results from Poland, Austria, and UK, mentioned above, are in accordance with the findings from another study by Ismail and her co-workers, who investigated eating habits and lifestyle behaviors not in Europe but among residents of the Middle East and North Africa during the lockdown in 2020 [2]. In this study, it was reported that the percentage of participants consuming five or more meals per day increased during the pandemic, that 48.8% and 32.5% of the participants did not consume fruits and vegetables on a daily basis, that 44.1% of participants consumed sweets or desserts at least once every day, 32.9% consumed salty snacks (chips, crackers, and nuts) daily and 22.5% consumed sweetened drinks at least once per day. These dietary habits indicate a pattern that would also promote weight gain and an increase in the prevalence of obesity.

From the studies addressed in a very interesting review that was recently published, ten of them reported an increase in snacking, and in six of them, the participants increased their number of meals during lockdown [3]. Interestingly, eleven studies reported beneficial changes in dietary habits, namely an increase in fresh produce and home cooking and a reduction in the consumption of comfort food and alcohol. On the other hand, nine studies found a reduction in fresh produce, and an additional six observed an increase in comfort foods including sweets, fried food, snack foods, and processed foods. Two studies reported an increase in alcohol consumption. In eight studies, an increase in body weight was reported and in seven, a reduction in physical activity was also observed.

Consequently, the study by Skotnicka and colleagues [1], published in *Nutrients*, adds to our knowledge about the changes in nutritional habits in the COVID-19 era, which could increase the risk of obesity and decrease the function of the immune system. These changes reported in this study, and in others [2,3], include increased meal frequency and snacking; increased ordering of readymade meals; increased consumption of sweets, alcohol, processed food, and sweetened drinks; and decreased physical activity. Taking also into account that COVID-19 and the measures to prevent and treat the disease have been present for two years, changes in dietary habits and physical activity may be long term and could adversely affect several health indices. On the other hand, it is equally important to point out that the study indicated some positive food-related behaviors developed during self-isolation, particularly those related to the quality and the manner of preparation [1].

Changes in dietary habits are not observed only in adults but also in children and adolescents. In a recent systematic review from our group, the majority of the studies included reported an increased consumption of fats and fast foods, as well as processed food, sweet and salty snacks, and sugar-sweetened beverages [4]. In addition, all studies in this systematic review reported a significant decrease in physical activity together with an increase in sedentary and screen time. Interestingly, in China, where the disease started from, according to Qiu his co-workers [5], 28.1% of the children with normal Body Mass Index (BMI) before the lockdown became overweight or obese, 42.4% of the overweight children became obese, and 46.6% of the children who started the study as normotensives experienced an increase in blood pressure at the end of the quarantine.

Changes in dietary habits and food consumption from 2020 up to now, as a result of the pandemic, would subsequently affect nutrient intake. In a very interesting review, Muscogiuri et al. [6] indicated that boredom, which is a natural consequence of quarantine, has been associated with a higher dietary energy, fat, carbohydrate, and protein intakes. Moreover, the continuous negative exposure to news on the pandemic from the media would increase stress and could lead people toward overeating, mostly looking for sugary “comfort foods”. It is known that carbohydrate cravings encourage the synthesis of serotonin, which would subsequently lead to a beneficial effect on mood. This unhealthy nutritional habit could increase the risk of developing obesity, which is a major risk factor of cardiovascular diseases, diabetes mellitus, and diseases of the respiratory system that are reported to increase the risk of serious complications of COVID-19. Increased consumption of fat, carbohydrates, and protein has often been observed to be positively associated with micronutrient deficiencies, which in turn could impair the function of the immune system. Several studies have reported that an increased consumption of fruits and vegetables, which are major sources of several important micronutrients, could increase the number of T-cell subsets, enhance lymphocyte response to mitogen, increase interleukin-2 production, and positively affect natural killer cell activity [6]. Finally, it has also been observed that vitamin D could have beneficial effects on the function of the respiratory tract, preserving tight junctions, killing enveloped viruses, and decreasing production of proinflammatory cytokines by the innate immune system, causing the reduction in the risk of a cytokine storm leading to pneumonia [6].

The question then arises as to whether a healthy diet is adequate, or if it is necessary to supplement it with several nutrients. To contribute to this scientific concern, Louca et al. [7] recruited 445,850 subscribers of an app that was developed to facilitate self-reported information related to SARS-CoV-2 infection. From these subscribers, 372,720 were in the UK, 45,757 in the USA, and 27,373 in Sweden. From the participants in the UK, 175,652 were consuming supplements and 197,068 were not. It was observed that those who were taking probiotics, omega-3 fatty acids, multivitamins, or vitamin D had a lower risk of SARS-CoV-2 infection by 14%, 12%, 13%, and 9%, respectively, after adjusting for potential confounders. The consumption of vitamin C, zinc, or garlic supplements had no effect risk of SARS-CoV-2 infection. Interestingly, the beneficial associations in individuals taking probiotics, omega-3 fatty acids, multivitamins, and vitamin D were observed only in females. The same overall pattern of association was observed in US and Swedish cohorts. Therefore, recommending vitamin and mineral supplements does not necessarily mean expectation of beneficial outcomes.

Finally, a recent review summarized nutritional guidelines to support dietary counseling provided by dietitians and health-related professionals [8]. In this review, it was reported that most of the guidelines recommended the consumption of fruits, vegetables, and whole grain foods. Thirty-one percent of the guidelines highlighted the importance of minerals and vitamins such as zinc and vitamins C, A, and D to maintain an efficient function of the immune system. On the other hand, it was also pointed out that dietary supplementation was not found to be associated with the prevention of COVID-19. However, it was also recommended that supplementation with vitamins C and D and with zinc and selenium could have some protective effects but only in patients with, or at risk of, respiratory viral infections or in patients with a detected nutrient deficiency. It should also be stressed that, even though some vitamins and minerals could improve immune system function, this should not lead to overconsumption. Intakes of megadoses can become toxic, induce various side effects, or even adversely interact with drug treatment.

In conclusion, the study by Skotnicka et al. [1] gives valuable information regarding the change in dietary habits and physical activity in European countries, as a result of the COVID-19 outbreak. These data, together with the results from other nutritional studies and in parallel with the vaccination programs, can be used to develop policies at national or European levels to prevent and treat the disease in the most probable efficient way.
